# Unlocking early academic skills: children’s cognitive processes, learning skills, and parental beliefs and behaviors predicting children’s language and math skills

**DOI:** 10.3389/fpsyg.2025.1610243

**Published:** 2025-08-20

**Authors:** Anne-Mai Meesak, Dmitri Rozgonjuk, Tiia Õun, Eve Kikas

**Affiliations:** ^1^School of Educational Sciences, Tallinn University, Tallinn, Estonia; ^2^Institute of Psychology, University of Tartu, Tartu, Estonia; ^3^School of Natural Sciences and Health, Tallinn University, Tallinn, Estonia

**Keywords:** early learning, academic skills, cognitive processes, learning skills, parental beliefs and behaviors, e-assessment

## Abstract

**Introduction:**

This study explored the emerging academic skills of five-year-old Estonian children, focusing on cognitive processes, learning skills, and parental beliefs and behaviors. While previous research has concentrated on a limited number of skill areas and aspects of the home environment, this study aimed to provide a more comprehensive understanding of children’s early learning by studying multiple skills and parental characteristics concurrently.

**Methods:**

Data was collected through direct e-assessments of children’s skills alongside parental questionnaires (*N* = 279).

**Results:**

We found positive relationships between children’s cognitive processes, language, math, and learning skills, emphasizing the importance of considering multiple skills together. While children’s cognitive processes and learning skills contribute to the development of their academic skills, parental beliefs and behaviors are also important. Considered as a whole, parental perceptions of their children’s cognitive difficulties and kindergarten involvement predicted both language and math skills, whereas parents’ education and social expectations were only related to language outcomes. In contrast, children’s learning skills predicted solely their math skills. Importantly, the frequency of parental home activities was not directly linked to children’s academic skills, showing that their relationship in early childhood might be more complex.

**Discussion:**

These results highlight the significance of a holistic approach to children’s development, integrating both child- and parent-related factors and suggesting that active participation in kindergarten and fostering social skills may outweigh high academic expectations and frequency of home-based activities in supporting children’s academic growth.

## 1 Introduction

Research has shown that children’s early learning experiences affect their later life (Bartik, 2014; Brownell and Drummond, 2020) and parents play a crucial role in ensuring children have the best possible start (Fagan et al., 2024; Haney and Hill, 2004; Sénéchal and LeFevre, 2002; Skwarchuk et al., 2014). Children’s learning often depends on verbal engagement with others, as their skills develop through social interactions with their family and peers (Vygotsky, 1978). Parental beliefs and behaviors in relation to their children are therefore especially important in early childhood, when children develop fundamental skills in the home environment (Shuey and Kankaraš, 2018; Skwarchuk et al., 2014). Perceptions of children’s characteristics (Phillipson and Phillipson, 2010) and expectations for their skills (Lai et al., 2022; Liu and Hoa Chung, 2022) are categorized as parental beliefs, while parental home activities (Haney and Hill, 2004; Skwarchuk et al., 2014) and kindergarten involvement (Manz et al., 2004; Puccioni, 2018) are categorized as parental behaviors.

Even though there is extensive research into children’s early learning (i.e., the period before formal education), most studies have concentrated on the relationship between cognitive processes and emerging academic skills (Best et al., 2011; Cameron et al., 2019; Morgan et al., 2019). Only a few studies have considered other developmental areas simultaneously, showing the contribution of learning skills to academic skill development (McClelland et al., 2006; Yen et al., 2004). While some studies have assessed children’s academic skills directly, learning skills have been mostly teacher assessed, with only a few exceptions (Meesak et al., 2022). There are also studies about the links between children’s skills and parental home activities (Casey et al., 2018; Haney and Hill, 2004; Skwarchuk et al., 2014), expectations of children’s skills (DeFlorio and Beliakoff, 2015; Liu and Hoa Chung, 2022), perceptions of children’s developmental indicators (Rutchick et al., 2009) or parental involvement in education (Hill and Taylor, 2004; Puccioni, 2018), but to our knowledge, none have combined all these aspects. This leaves room for further studies in early learning and the relationships with parental beliefs and behaviors. In order to get a holistic view of the development of children’s academic skills, various skill areas and aspects of parental beliefs and behaviors should be examined concurrently.

The aim of the present work is to examine the emerging academic skills of five-year-old Estonian children in relation to their cognitive processes and learning skills and parental beliefs and behaviors. It is important to gather information about children’s skills and aspects supporting and hindering them as early as age five, as most children attend kindergarten by that time (Statistics Estonia, 2024), making it possible to improve their skills before they start primary education.

First, we study the contribution of five-year-old children’s cognitive processes and learning skills to their language and math skills development. Second, we determine the characteristics of parental home activities, expectations of children’s skills needed for starting primary education, kindergarten involvement and perceptions of children’s characteristics, alongside children’s cognitive processes and learning skills predicting their language/math skills. The current study offers insight into the development of children’s early academic skills and the child- and parent-related characteristics supporting or hindering them. The results can be used to inform kindergartens in setting goals for five-year-old children’s skills, raise parents’ awareness of their perceptions and expectations of their children, provide information about parental home- and kindergarten-related behaviors, which are most beneficial to children, and promote cooperation between parents and kindergartens.

### 1.1 Children’s early learning

Learning is a life-long process, which begins long before children can grasp the meaning of the term. ‘Early learning’ is a term used for the period when children do not yet attend primary education, but nevertheless have started their learning journey (Shuey and Kankaraš, 2018). Research has shown the importance of children’s early learning in relation to their later academic success (Aunio and Niemivirta, 2010; Brownell and Drummond, 2020; Morgan et al., 2019). Researchers have concentrated on early learning domains such as physical, cognitive, social and emotional, language and math skills, and learning behaviors as they are critical for children’s development (Pisani et al., 2018; Schoon et al., 2015; Shuey and Kankaraš, 2018; Yen et al., 2004).

Most studies about children’s early learning have shown the importance of developing cognitive processes and academic skills (Best et al., 2011; Cameron et al., 2019; Fuhs et al., 2014; Morgan et al., 2019). Cognitive processes include working memory, cognitive or mental flexibility, inhibitory control, attention and perceptual skills, among others (Cameron et al., 2019; Shuey and Kankaraš, 2018), while ‘early academic skills’ usually refers to emerging literacy and numeracy or language and math (Cameron et al., 2019; Morgan et al., 2019). Cameron et al. (2019) studied relations between children’s cognitive and academic skills before and after kindergarten. They found evidence for the co-development of cognitive and academic skills during kindergarten – improvements in one skill area happened simultaneously with improvements in others (Cameron et al., 2019). Morgan et al. (2019) examined the role cognitive deficits play in children’s risk of experiencing difficulties in academic domains across elementary school. They found that deficits in working memory, in particular, increased the risk of having difficulties in reading, math and science, while deficits in cognitive flexibility and inhibition had less predictive power (Morgan et al., 2019).

Less often, studies have involved learning skills, which also play a crucial role in academic skill development and encompass behaviors and competences related to self-regulation, persistence, motivational effectiveness and approaches to learning (McClelland et al., 2006; Yen et al., 2004). McClelland et al. (2006) and colleagues provided evidence of the importance of developing learning-related skills as early as the kindergarten, as they were related to reading and math, not only in kindergarten, but also in predicting academic outcomes in primary school. Similarly, Yen et al. (2004) showed that 6-17-year-old children’s learning behavior made a unique contribution to their academic outcomes. Even though both of these studies brought attention to the importance of learning skills in helping to achieve academic success, teachers indirectly assessed these skills; however, previous studies have shown discrepancies between children’s outcomes and teachers’ ratings (Kowalski et al., 2018; Waterman et al., 2012; Vitiello and Williford, 2021), which would make direct assessment preferable. Theories of self-regulated learning (Boekaerts, 1997; Zimmerman, 2002) and expectancy-value of achievement motivation (Eccles et al., 1983; Wigfield and Eccles, 2000) are widely used to frame learning skills. Drawing from these established theories, the current research focuses on a limited set of age-appropriate constructs of learning skills – children’s interest, reflecting the enjoyment and value they place on learning tasks (Eccles and Wigfield, 2002), self-efficacy, referring to their belief in their ability to succeed (Bandura, 1997), and self-confidence, capturing their perceived competence of completing tasks (Zimmerman, 2002). Previous research has shown that children’s interest (Doctoroff et al., 2016) and self-beliefs (Valentine et al., 2004) lead to higher academic achievement.

There are no widely available and suitable assessment instruments, which cover various skill areas (Pisani et al., 2018; Reid et al., 2014), even though previous research has shown that even direct e-assessment is appropriate for young children (Adkins, 2021; OECD, 2020b). For the current study, data regarding children’s cognitive processes, language, math, and learning skills is gathered using a directly administered standardized test (Meesak et al., 2022), allowing us to simultaneously obtain information regarding various crucial skill areas, while avoiding possible rater bias.

### 1.2 Parental beliefs and behaviors in relation to children’s skills

According to Bronfenbrenner’s (1979) ecological systems theory, children’s development is shaped by multiple interconnected systems. These systems include immediate contexts (e.g., home or kindergarten), relationships between environments (e.g., kindergarten-based parental involvement), aspects not directly related to children (e.g., parental occupation), broader cultural, social and economic contexts, and changes happening over time (Bronfenbrenner, 1979). Understanding the interactions among the systems can provide insights into children’s holistic development. According to Vygotsky (1978), children’s learning and cognitive development are facilitated through social interactions in cultural contexts. Vygotsky (1978) and Bronfenbrenner (1979) both underscore the interplay between environmental factors and children’s development, highlighting the significance of social interactions, cultural and ecological contexts in shaping cognitive, social, and emotional growth.

Researchers have increasingly acknowledged the importance of the home environment and parental beliefs and behaviors in relation to children’s early learning outcomes, showing that Vygotsky’s (1978) and Bronfenbrenner’s (1979) theories are still relevant (Casey et al., 2018; Daucourt et al., 2021; Frédérique et al., 2024; Liu and Hoa Chung, 2022; Liu et al., 2020; Phillipson and Phillipson, 2010). Studies have shown that parental beliefs regarding their children’s characteristics (Rutchick et al., 2009) and skills needed for starting primary education (Lai et al., 2022; Liu and Hoa Chung, 2022; Slicker et al., 2021) are related to early skill development. Similarly, parental behaviors such as home activities or kindergarten involvement can support or hinder children’s skills in various areas (Casey et al., 2018; Haney and Hill, 2004; Hill and Taylor, 2004; Skwarchuk et al., 2014). Previous research about parental behaviors and children’s skills have mostly concentrated separately on the benefits of parental home activities for children’s academic skills in emerging literacy (Haney and Hill, 2004; Lai et al., 2022; Sénéchal and LeFevre, 2002) or numeracy (Casey et al., 2018; Elliott and Bachman, 2017), though a few exceptions have included both areas (Fagan et al., 2024; Skwarchuk et al., 2014). Similarly, studies have shown the relationship between parental expectations and children’s cognitive and language skills (Liu and Hoa Chung, 2022). There is some research into the associations of children’s academic outcomes with parental expectations and school-based involvement (Hill and Taylor, 2004; Puccioni, 2018) and parental expectations and home activities simultaneously (DeFlorio and Beliakoff, 2015; Skwarchuk et al., 2014; Slicker et al., 2021), but the results have been somewhat inconsistent. Skwarchuk et al. (2014) found that informal parental home practices predicted children’s non-symbolic arithmetic and vocabulary, while formal practices did not. In contrast, symbolic number knowledge and letter-word reading were predicted by formal practices (Skwarchuk et al., 2014). Haney and Hill (2004) studied the relationship between parental home teaching activities and children’s emergent literacy. They found that teaching alphabet sounds is related to children’s vocabulary (receptive and expressive), while teaching writing words is related to alphabet knowledge (letters and theirs sounds). On the other hand, teaching literacy skills, letter names, printing letters, reading words, reading stories was not related to either vocabulary, alphabet or conventions (understanding of English print).

There are also studies, which have not found direct links between home activities and children’s outcomes (DeFlorio and Beliakoff, 2015; Missall et al., 2015). DeFlorio and Beliakoff (2015) found that children’s numeracy outcomes were related with parental expectations of children’s mathematical ability, but not with children’s home activities. However, Missall et al. (2015) found no evidence of a relationship between children’s early math performance relations and either math beliefs or math activities. In the previously mentioned study by Skwarchuk et al. (2014), parental attitudes to numeracy predicted children’s outcomes, while attitudes to literacy and expectations did not.

In 2018, OECD arranged the International Early Learning and Child Well-being Study (IELS) in Estonia, England and the United States. The study found relationships between children’s emergent academic skills and their perceived difficulties, some home activities, the number of children’s books at home and parental involvement with the kindergarten (OECD, 2020b). In examining the combined effects of child and parental characteristics on children’s academic results in Estonia, they found children’s age, socioeconomic status, the language of the kindergarten, the number of children’s books and parental perceptions of children’s learning difficulties to be significant predictors of children’s academic skills. Math home activities and parental kindergarten involvement were only related to math skills, while the child’s gender, home language and being read to at least five times a week were related to language skills. Other home activities, such as having conversations, telling stories, etc., did not emerge as significant (OECD, 2020a).

The somewhat inconsistent results discussed above indicate that young children’s outcomes and parental relations should be further analyzed to determine the specific characteristics contributing to children’s early academic skill development. The current study expands the existing knowledge of children’s emerging language and math skills by examining the contributions of children’s cognitive processes and learning skills alongside parental perceptions of their children’s characteristics, expectations of children’s skills, kindergarten involvement and home activities.

### 1.3 Context of the study

Estonia is a small Baltic country, with a total population of approximately 1.3 million, of which 5% were five- to-nine-year-olds in 2021 (Statistics Estonia, 2024). Estonia consistently ranks among top-performing countries in the OECD Programme for International Student Assessment (PISA), particularly in reading and math (OECD, 2019, 2023), with quality early childhood education being credited as one of the reasons behind students’ success (Education Estonia, 2023; Eisenschmidt et al., 2023). In Estonia, attending early childhood education is voluntary, but all children between 18 months and seven years are legally entitled to a place in a kindergarten, for which fees cannot exceed 20% of the minimum wage (OECD, 2020a). Studies have shown that Estonia has been quite successful in mitigating the differences in socio-economic background, with children from higher and lower groups having only small differences in academic outcomes (OECD, 2020b). The work of all kindergartens is based on the National Curriculum for Preschool Child Care Institutions, which emphasizes play-based learning and supports holistic development of children by valuing both general skills such as cognitive and learning skills, next to academic skills and subject areas like language and math (The Estonian Government, 2008/2011). According to official statistics, 96% of five-year-old children attended kindergartens in 2021 (Statistics Estonia, 2024).

Estonia has been regarded as a leader in digital transformation in education, with early integration of digital tools in learning environments since the “Tiger Leap” in 1997, which helped to provide computers to schools (Vitureau, 2023). The following “ProgeTiger” program guaranteed access to high-quality ICT education beginning from early childhood education with teachers applying various digital tools to enrich learning (Education Estonia, 2021) and by 2023, 79% of all educational institutions in Estonia had participated in some of its activities (Õunapuu et al., 2023). In the OECD (2020b) IELS study, Estonian parents reported that 91% of their five-year-old children used a digital device at least once a month, which was slightly less than in England (94%) and the United States (95%). According to the study, five-year-old children in Estonia and England did similarly well in language skills, while Estonian children were overall better in cognitive processes and socio-emotional skills. English children had somewhat better math skills, while children in the United States scored overall lower in all areas. Even though five-year-old Estonian children achieved good results in the international study (OECD, 2020b), no official expected outcomes are specified for this age group in the national curriculum and no compulsory testing taking place in kindergarten (The Estonian Government, 2008/2011). However, since the beginning of 2023, all kindergarten teachers and specialists can use an e-assessment instrument consisting of three tests and introductory items to assess five-year-old children’s skills. The tests were developed in cooperation between the Estonian Education and Youth Board and experts in psychology, educational technology, early childhood and special education from Estonian universities (Kikas et al., 2022), drawing on the experience of participating in the OECD (2020a) IELS study.

### 1.4 The present study

**Figure 1:**
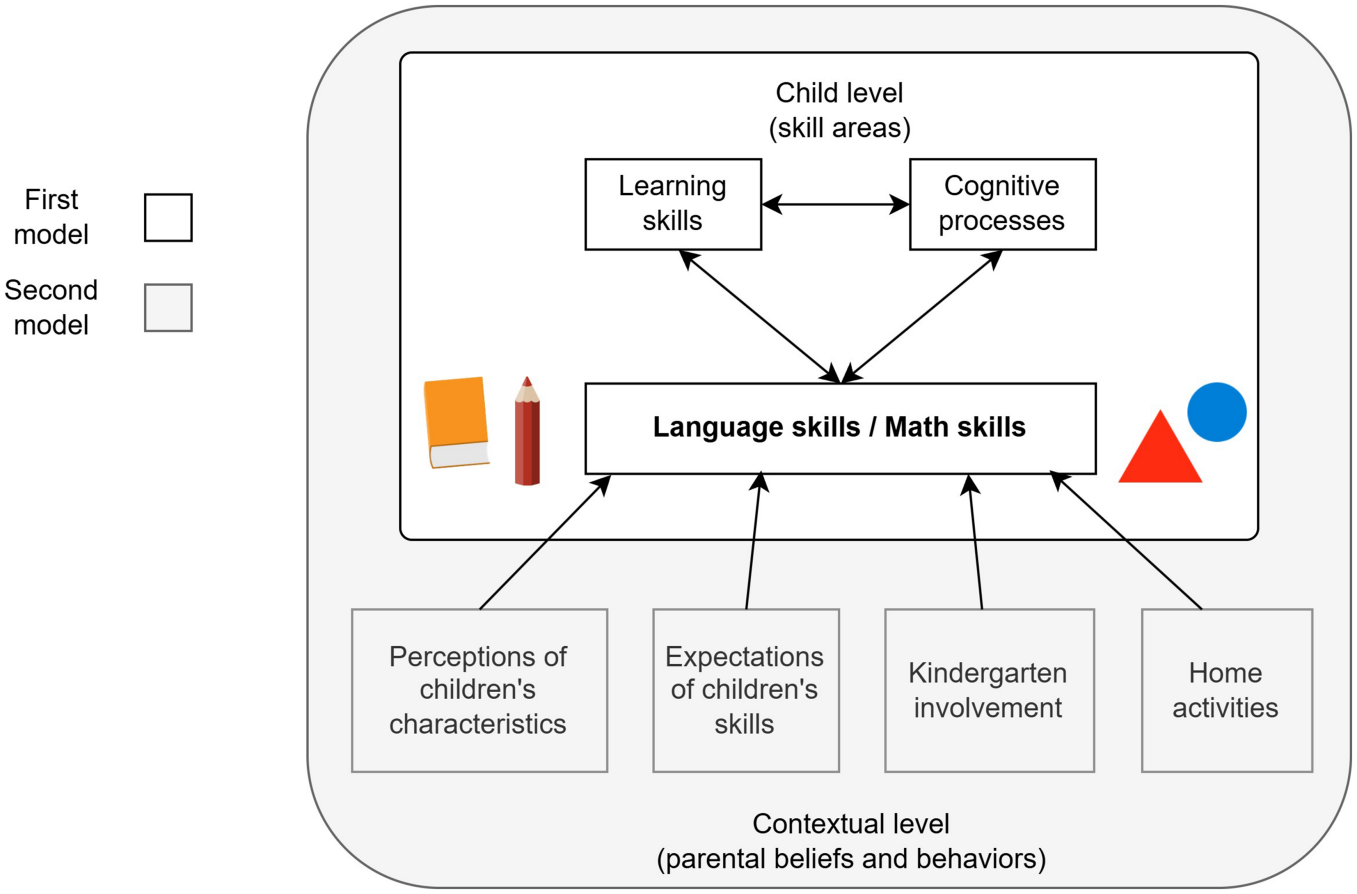
Hierarchical models for children’s language/math skills development.

The aim of the present study is to examine the emerging academic skills development of five-year-old Estonian children in relation to their cognitive processes and learning skills and their parents’ beliefs and behaviors. The parental beliefs and behaviors considered include parental perceptions of their children’s characteristics, expectations of children’s skills needed for starting primary education, kindergarten involvement and home activities. The present work allows us to investigate the potentially interrelated effects of predictors on children’s early academic outcomes, while also investigating specific characteristics of child-assessed and parent-related variables in terms of academic skills development. While children’s skills are directly assessed, the study focuses on self-reported parental beliefs and behaviors without examining their quality. Hierarchical models are constructed separately for language and math skills. The theoretical models are shown in Figure 1. First, a focus is set on children’s cognitive processes and learning skills in relation to their emerging language/math skills. Second, the characteristics of parental perceptions, expectations, kindergarten involvement and home activities are included in the models.

The present study focuses on the following research questions (RQs) and hypotheses (Hs):

(RQ1) How are children’s cognitive processes and learning skills associated with language and math skills?

(*H1*) *Children’s cognitive processes, language, math, and learning skills are all positively related.* Previous research has shown that children’s skills in different areas have moderate positive associations (Best et al., 2011; Cameron et al., 2019; McClelland et al., 2006; Meesak et al., 2022). The relationship has been shown to be strongest for academic skills and somewhat lower for learning skills (Meesak et al., 2022; Yen et al., 2004). This is in the nature of a confirmatory hypothesis.

(RQ2) Which aspects of children’s cognitive processes and learning skills together with parental beliefs and behaviors predict children’s language and math skills?

(*H2*) *Children’s cognitive processes and learning skills, parental home activities, perceptions of their children’s characteristics, kindergarten involvement and expectations of the skills needed to start primary education predict children’s language* (H2a) *and math* (H2b) *skills development.* Studies have separately shown the contributions of children’s cognitive processes (Best et al., 2011; Fuhs et al., 2014) and learning skills (McClelland et al., 2006), parental home activities (Elliott and Bachman, 2017; Haney and Hill, 2004; OECD, 2020a), expectations (Skwarchuk et al., 2014; Slicker et al., 2021) and school-based involvement (OECD, 2020a; Puccioni, 2018) in children’s academic skills development.

## 2 Methods

### 2.1 Sample and procedure

The study was part of a larger investigation conducted by the Estonian Education and Youth Board on behalf of the Estonian Ministry of Education and Research. The current study was conducted in 46 kindergartens in Estonia at the end of 2021. The study involved five-year-old children, their parents, and teachers. Probability sampling using multiple probability techniques was used to obtain the sample. First, 50 kindergartens with at least 10 five-year-old children were sampled across Estonia, with the language of instruction as stratification. In Estonia, in addition to Estonian-language kindergartens, there were kindergartens with Russian as the language of instruction (until Sept 2024). All of the kindergartens adhere to the National Curriculum for Pre-school Child Care Institutions (The Estonian Government, 2008/2011).

Initially, the sample comprised of 42 Estonian-language kindergartens and eight Russian-language kindergartens, each of which received an invitation to participate in the study. If a kindergarten did not respond or declined to participate, a replacement kindergarten was selected. In the final sample, there were 46 kindergartens (38 Estonian-language and eight Russian-language). Next, 10 children born between 1st October 2015 and 1st November 2016 were sampled from each kindergarten. As the study was commissioned by the Estonian Ministry of Education and Research and carried out by Education and Youth Board, an additional approval from the ethics committee was not required. Education and Youth Board is the governmental authority responsible for national external educational assessments in Estonia according to its statutes (Minister of Education and Research, 2020). However, the study followed the American Psychological Association’s ethical guidelines. Parents were sent an extensive information letter informing them about the study’s aims, procedure and ethical aspects, together with a consent form for their child’s participation. Their right to withdraw their consent at any stage of the study was explained, among other matters. The parents were also informed that participation involved filling out a parental questionnaire. Parents gave their informed consent for their child’s participation, and each child was also asked to give their verbal consent to participate, due to their age, by playing a game on a tablet. Opt-in consent was considered necessary for participation in all stages.

Children’s skills in four areas were assessed using a tablet computer, with each child receiving individual assistance from a staff member with pedagogical competences (e.g., teacher, special education specialist). Since the assessment could be stopped as needed, each child participated in several assessment sessions. Study administrators received comprehensive training, including an extensive manual and online sessions. The manual covered assessment details, procedures, introductory item screenshots, and specific suggestions. Administrators were trained to familiarize children with characters and question types, provide encouragement, and offer instructions during introductory items. During the assessments, only technical support was allowed (e.g., “Push this button to hear the instructions”). In total, 397 five- to six-year-old children (age at start of testing period M = 64.55 months, SD = 3.31 months) participated in the study. A little more than a half (52%) were girls, 78% solved the test in Estonian (*N* = 309) and 22% in Russian (*N* = 88). The testing period was from 1st October to 15th December 2021.

The parents filled out an online questionnaire, which was available in Estonian and Russian. In total, 308 parent questionnaires were completed. The majority (77%, *N* = 237) of parents filled out the questionnaires in Estonian (*N* = 237), while a fourth (23%, *N* = 71) filled out the Russian version. There were 248 mothers, 14 fathers and 46 mother–father dyads in the sample. The mothers were on average 36 years old (M = 35.62; SD = 5.14) and fathers were on average 37 years old (M = 37.07; SD = 5.73). Three quarters of the mothers (75%, *N* = 217) reported talking with their children in Estonian and a quarter (25%, *N* = 70) in Russian. One mother reported being bilingual. The majority (81%, *N* = 48) of the fathers reported talking in Estonian, others in Russian (19%, *N* = 11). The questionnaires were completed online in LimeSurvey between 1 October and 15 December 2021.

Due to incomplete questionnaires from some parents and because some children did not participate in assessments although their parents completed the questionnaire, answers from 279 children (55% girls, 79% in Estonian) and their parents were included in the study. A little more than a half (54%) of the children were girls, 79% solved the test in Estonian (*N* = 221) and 21% in Russian (*N* = 58).

### 2.2 Measures

#### 2.2.1 Children’s test

A test belonging to an e-assessment instrument developed by the Estonian Education and Youth Board and Tallinn University was used to measure children’s skills (Kikas et al., 2022). The instrument was developed based on the preschool curriculum (The Estonian Government, 2008/2011) and previous studies (Männamaa and Kikas, 2011; OECD, 2020b). The e-assessment instrument is standardized and its structure has been confirmed using confirmatory factor analysis (CFA) in two previous studies (Meesak et al., 2022, 2025). The results of the test have been validated using teachers’ evaluations – direct assessment results of constructs of children’s cognitive processes, language and math skills largely coincided with teachers’ evaluations (*r* = 0.35–0.77), except for learning skills (Meesak et al., 2022), which can be attributed to teachers’ having difficulties accurately evaluating children’s learning behaviors (Baroody and Diamond, 2013). It has been noted that e-assessment offers possibilities for making the testing more engaging for children (Adkins, 2021), while direct assessments help to reduce the risk of rater bias (Waterman et al., 2012; Vitiello and Williford, 2021). For this reason, all items include original illustrations and audio instructions, have child-friendly design and are entirely computer-assessed.

**Figure 2:**
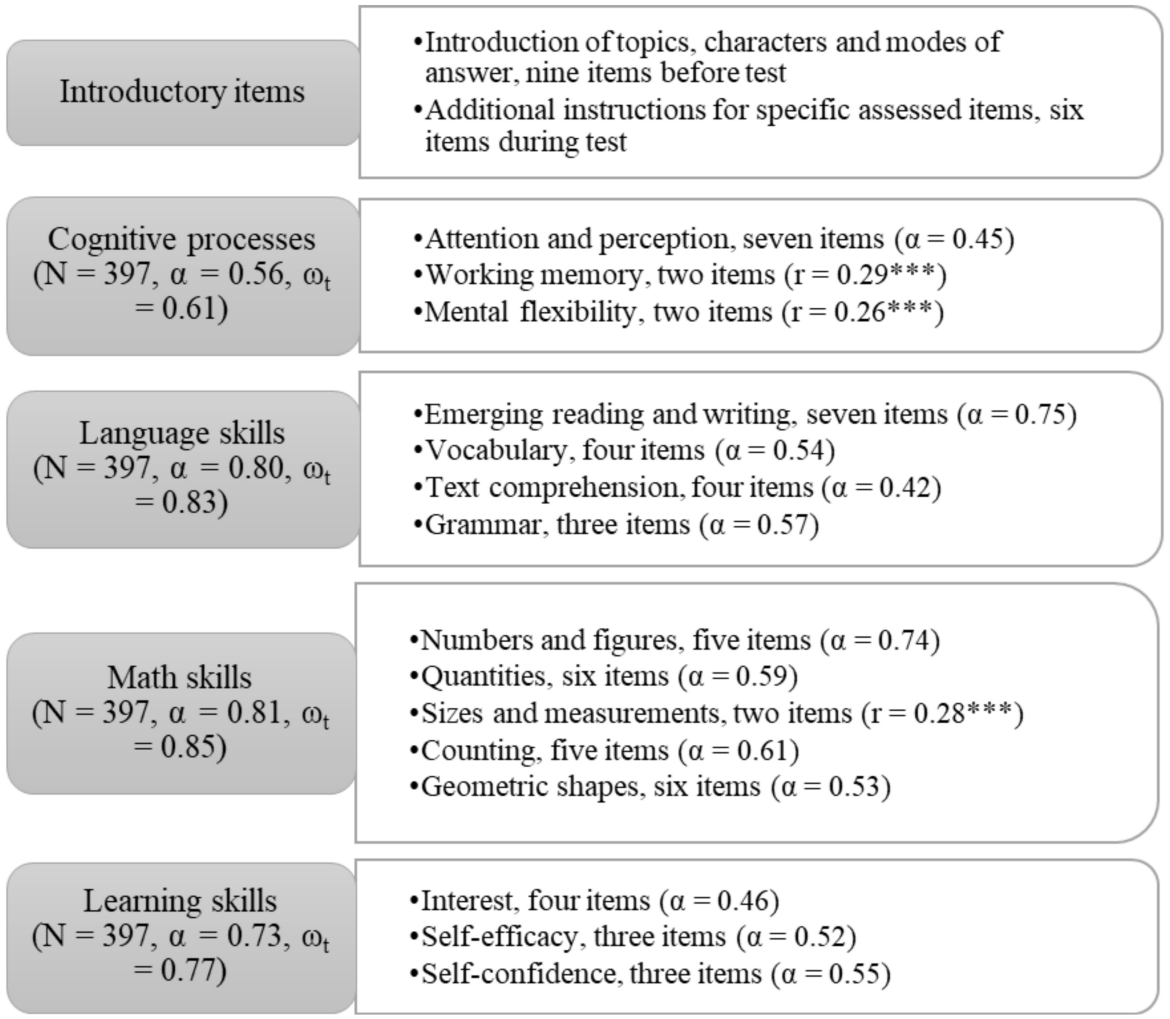
Structure of the children’s test. α, Cronbach’s alpha; ω_t_, McDonald’s total omega; *r*, Pearson’s correlation; ***p < 0.001.

The implemented test assesses children’s four developmental areas: cognitive processes, language, math, and learning skills, with a consistent theme of “children at home” integrating these areas. The test includes 63 assessed items and six non-assessed items. Additionally, nine introductory items precede the test, introducing themes, characters, question types and response modes (touch, drag and drop). The structure of the children’s test, including the constructs in each area and their reliability statistics, are shown in Figure 2. Three constructs are assessed in children’s cognitive processes: attention and perception (finding similarities and differences in pictures, comparing objects, listening and comparing sounds), working memory (changing rules) and mental flexibility (sequencing pictures). The language skills include four constructs: emerging reading and writing (letter recognition and phoneme analysis), vocabulary (general terms and opposite words), text comprehension (making conclusions based on text) and grammar (formation of impersonal form). Five constructs are assessed for the math skills: numbers and figures (connecting number signs with figure names, ordering figures), quantities (comparing quantities), sizes and measurements (comparing objects), counting (counting objects and sounds) and geometric shapes (knowing geometric shapes and objects). For learning skills, child’s interest (indicating liking something), self-efficacy (indicating their ability level) and self-confidence (indicating their certainty of their answer) are assessed. Four-factor CFA showed an acceptable model fit, scaled *χ*^2^ (84) = 202,787, *p* < 0.001, robust CFI = 0.91, robust TLI = 0.88, robust RMSEA = 0.06 [90% CI: 0.05, 0.07], SRMR = 0.05.

The test was first developed in Estonian, then adapted to the Russian language by a language expert, then back-translated and adapted again in Russian. The configural, metric and scalar invariance has been tested and the factorial structure of the e-assessment instrument has been confirmed to be similar across Estonian and Russian versions in a previous study (Meesak et al., 2025). In the current study, the metric invariance of the test was established across language groups, which is sufficient to justify pooling data for regression analyses (Chen, 2007); specifically, the configural model showed acceptable fit (CFI = 0.89, RMSEA = 0.07, SRMR = 0.06), and the metric model demonstrated comparable fit (CFI = 0.89, RMSEA = 0.07, SRMR = 0.06), with changes in fit indices (ΔCFI = 0.003, ΔRMSEA = −0.003, ΔSRMR = 0.004) remaining within recommended thresholds.

#### 2.2.2 Parental questionnaire

The information regarding parental beliefs and behaviors was gathered with a questionnaire. The questionnaire was developed based on previous research and included four thematic blocks. The first block included parental perceptions of children’s characteristics: 19 statements about social (e.g., *Difficulty understanding explanations and instructions*) and cognitive difficulties (e.g., *Difficulty judging direction and spatiality*) and 11 statements about children’s learning (e.g., *The child completes started activities*) and disruptive (e.g., *The child behaves impulsively*) behaviors (Kadesjö et al., 2017; Tucker-Drob and Harden, 2012, 2013). All statements were evaluated on a five-point scale (1 = never, 2 = rarely, 3 = sometimes, 4 = often, 5 = very often). The statements about positive learning behavior were reverse coded in the data analysis stage to indicate learning avoidance.

The second block included 14 statements about parental language (e.g., *Ask to point to words or letters while reading*), math (e.g., *Play with numbers or count with my child*), and social skill related (e.g., *Play creative role games*) home activities (Kikas et al., 2011; OECD, 2020b; Skwarchuk et al., 2014; Slicker et al., 2021). All statements were evaluated on a five-point scale (1 = never, 2 = less than once a week, 3 = 1–2 times a week, 4 = 3–4 times a week, 5 = 5–7 times a week).

The third block included seven statements about parental involvement in kindergarten activities, divided into kindergarten-based involvement (e.g., *Help the teacher to plan events*) and home-kindergarten conferencing (e.g., *Talk to the teacher about what the child should practice at home*) (Fantuzzo et al., 2006; Kikas et al., 2011; Manz et al., 2004). The scale of the first block was used.

The last block included 19 statements about parental expectations of the academic (e.g., *The child can count to 12*), learning (e.g., *The child is able to plan their own learning and the strategies used*), and social skills (e.g., *The child is able to consider others and cooperate*) needed for starting primary education (Skwarchuk et al., 2014; The Estonian Government, 2008/2011). All the statements were on a five-point scale (1 = not important, 2 = rather not important, 3 = neutral, 4 = rather important, 5 = very important).

**Figure 3:**
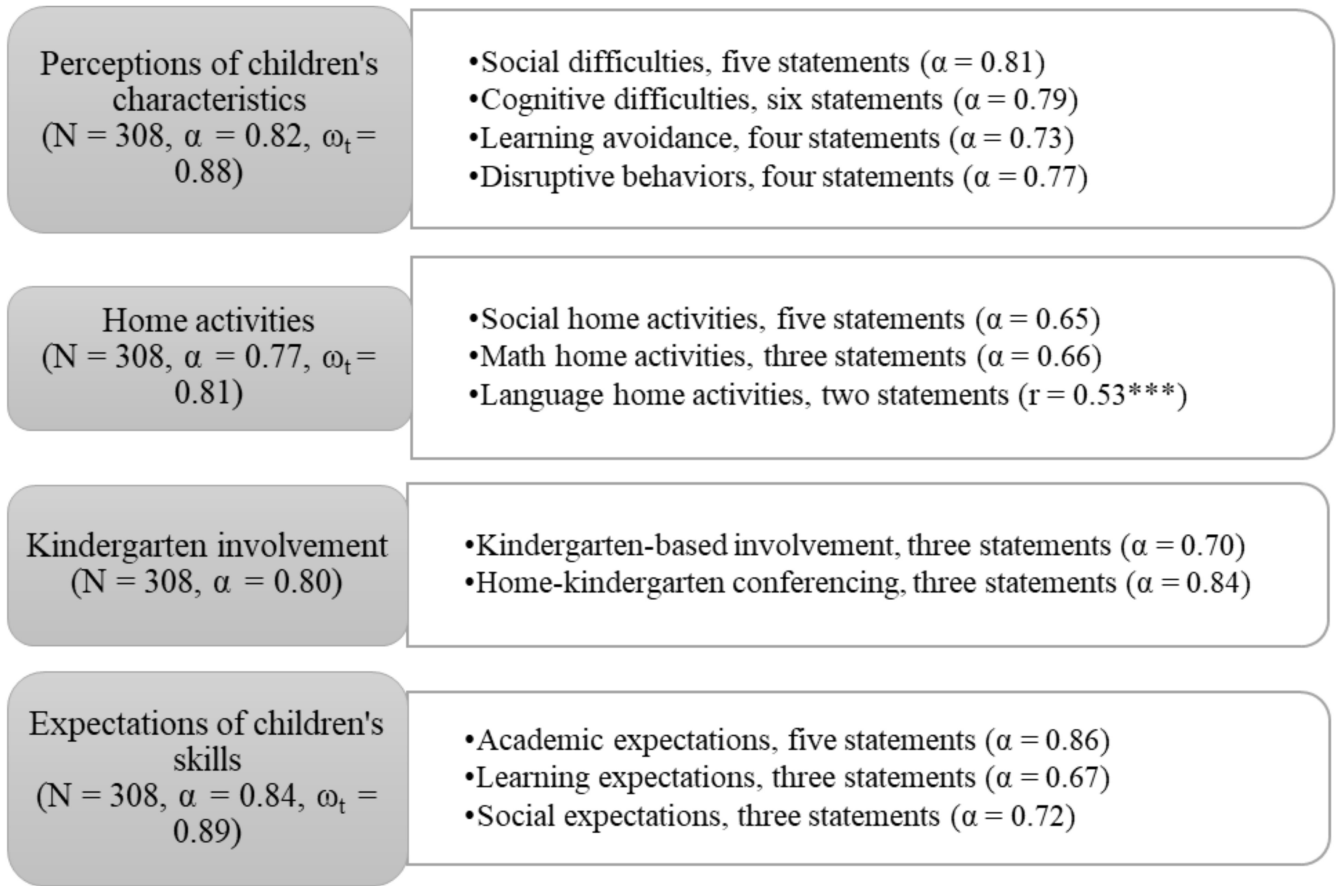
Structure of the parental questionnaire. α, Cronbach’s alpha; ω_t_, McDonald’s total omega; *r*, Spearman’s correlation; ***p < 0.001.

Feedback for the questionnaire was obtained from experts in the fields of psychology and education. The questionnaire was developed in Estonian and then translated into Russian. The translation was reviewed by one Russian language expert and one native speaker. The structure of the questionnaire blocks along the configural and metric invariance of the structure across Estonian and Russian versions was confirmed (Appendix A). The final structure of the questionnaire, together with reliability statistics, is shown in Figure 3.

### 2.3 Data analysis

Data analysis was conducted using R version 4.3.1 (R Core Team, 2023). The reliability of the children’s test and parental questionnaire was first tested using Cronbach’s alpha (Cronbach, 1951) with the *alpha()* function and McDonald’s omega (McDonald, 1999) with the *omega()* function from the [psych] package v[2.2.5] (Revelle, 2022). Omega is reported for skill areas alongside alpha, as recent papers have argued for its advantage for calculating reliability (Goodboy and Martin, 2020; Kalkbrenner, 2023). Coefficients exceeding 0.65 are usually considered to have good internal reliability (Kalkbrenner, 2023; Nunnally, 1967), while with a limited number of items, acceptable coefficients can be lower (Cronbach, 1951). Next, confirmatory factor analysis (CFA) from the [lavaan] package v[0.6–12] with the Maximum Likelihood Robust Estimation Method (Rosseel, 2012) was used to examine the structure of the children’s test and parental questionnaire. In addition to chi-square (χ2), which non-significant value is usually not reached (Steiger, 2007), other common model fit benchmarks were used. According to Hu and Bentler (1999), acceptable comparative fit index (CFI) and Tucker-Lewis index (TLI) values should be at least 0.90, while acceptable standardized root mean square residual (SRMR) value should be below 0.08. According to MacCallum et al. (1996) acceptable root mean square error of approximation (RMSEA) value should also be below 0.08. The configural and metric invariance of the children’s test and parental questionnaire was tested using the [semTools] package v[0.5–6] (Jorgensen et al., 2022). Based on the recommendation of Chen (2007), CFI, RMSEA and SRMR changes were reviewed because of the sensitivity of chi-square tests to sample size and violation of assumption of normality. When sample sizes are small and unequal, metric invariance is supported if ΔCFI ≤ 0.005, accompanied by either ΔRMSEA ≤ 0.010 or ΔSRMR ≤ 0.025 (Chen, 2007).

The children’s assessment data was then linked with their parents’ answers and imputed using classification and regression trees from the [mice] package v[3.14.0] (van Buuren and Groothuis-Oudshoorn, 2011). Based on the sums of scores for the children’s assessment areas, Pearson correlations were calculated using the *corr.test()* function of the [psych] package (Revelle, 2022) to show the relationships between different areas of children’s assessment. The *p*-values were adjusted using Holm’s method, which protects against making a Type 1 error (Holm, 1979). Similarly, the correlations were calculated to show the relationship of children’s language and math skills to the constructs of children’s cognitive processes and learning skills, and the parental perceptions, home activities, kindergarten involvement and expectations. Guidelines by Gignac and Szodorai (2016) were used to interpret the coefficients: *r* < 0.10 very small, 0.10 ≤ *r* < 0.20 small, 0.20 ≤ *r* < 0.30 moderate, *r* ≥ 0.30 large.

Hierarchical multiple regression models were created to determine the specific constructs of children’s cognitive processes and learning skills, alongside parental perceptions, expectations, involvement and home activities, predicting children’s language and math skills. The ‘Enter’ method and the functions *stepwise()* and *lm()* from the [stats] package were used. The analysis of variance (ANOVA) test with the function *anova()* was used to compare the models. A leave-one-out-cross-validation (LOOCV) error diagnostic was finally performed, using the *hatvalues()* function to determine a final model.

## 3 Results

### 3.1 Children’s academic skills in relation to their cognitive processes and learning skills

To answer our first research question, we conducted a bivariate correlation analysis for children’s skill areas. We found that children’s language and math skills are positively related with each other and with children’s cognitive processes and learning skills (Table 1). The relation is the strongest between the two academic skills, while the associations between cognitive processes and language and math skills are also showing large effects. Although there is a weak relation between cognitive processes and learning skills, it becomes non-significant when adjusting the p-values by Holm’s method.

**Table 1:** Descriptive statistics and correlations for children’s skill areas.

Skill area	*N*	M	SD	Min	Max	Range	*r* with language skills	*r* with math skills	*r* with learning skills
Cognitive processes	279	34.77	3.47	21.50	40.50	0.00–40.50	0.47***	0.47***	0.17
Language skills	279	26.94	5.49	9.00	34.00	0.00–34.00	1	0.60***	0.21*
Math skills	279	54.02	6.05	21.50	60.00	0.00–60.00		1	0.27***
Learning skills	279	27.62	2.86	15.00	30.00	10.00–30.00			1

N, sample size; M, mean; SD, standard deviation; Min, observed minimum score; Max, observed maximum score; Range, theoretical range; *r*, Pearson correlation coefficient; *p*-values are adjusted by Holm’s method. ***p < 0.001, *p < 0.05.

### 3.2 Child-assessed and parent-related characteristics predicting children’s language and math skills

To answer our second research question, the specific constructs of children’s cognitive processes and learning skills were determined, alongside parental perceptions, expectations, kindergarten involvement and home activities predicting language and math skills. The descriptive statistics and correlation matrix are included in Appendices B, C. For both language and math skills, hierarchical multiple regression was used to make a distinction between child-assessed and parent-related predictors. For the first models, child-assessed predictors were added to the model with the forward stepwise selection method. Similarly, the second models were created with parental predictors (Table 2).

**Table 2:** Hierarchical regression analysis results of children’s academic skills.

Child-assessed predictors	Children’s skill area				
	Language skills		Math skills		
	Model 1 β	Model 2 β	Model 1 β	Model 2 β	Model 3 β
Attention and perception	0.37***	0.36 ***	0.31***	0.31***	0.31***
Mental flexibility	0.18***	0.17**	0.14**	0.13*	0.13*
Working memory	0.11*		0.24***	0.22***	0.22***
Self-efficacy	0.12*	0.07	0.12*	0.11*	0.12*
Self-confidence		0.08	0.12*	0.14*	0.14*
Parental predictors					
Perception of cognitive difficulties		−0.14**		−0.12*	−0.14**
Perception of disruptive behaviors		−0.07			
Social expectations		0.13*			
Learning expectations		0.09			
Kindergarten-based involvement		0.16**		0.12*	0.11*
Home-kindergarten conferencing		−0.12*			
Math-related home activities				0.07	
Parental education		0.12*			
Model *R*^2^	0.24***	0.35***	0.29***	0.33***	0.32***

Standardized coefficient estimates are shown (β). *p < 0.05, **p < 0.01, *** *p* < 0.001.

ANOVA was used to compare the two models. For the language skills, Model 1 (*R*^2^ = 0.24, *p* < 0.001) yielded a residual sum of squares (RSS) of 210.36, while Model 2 (*R*^2^ = 0.35, *p* < 0.001) showed a significantly lower RSS of 181.37, ΔF(7, 267) = 6.10, *p* < 0.001, indicating a better fit to the data and suggesting that the extended model accounts for significant variance in children’s language skills (Δ*R*^2^ = 0.11). Additionally, LOOCV error coefficients were calculated for Model 2 and a third model, leaving out all insignificant predictors, with results showing a preference for Model 2.

For the math skills, Model 2 (*R*^2^ = 0.33, *p* < 0.001) also showed a notably lower RSS of 186.80 compared to Model 1 (*R*^2^ = 0.29, *p* < 0.001) RSS of 197.77, ΔF(3, 270) = 5.29, *p* < 0.01, suggesting an improved model fit. LOOCV error coefficients were calculated for Model 2 and Model 3 leaving out all insignificant predictors (in this instance, only math-related home activities), with Model 3 (*R*^2^ = 0.32, *p* < 0.001) being slightly preferable in this case (Δ*R*^2^ = 0.03).

## 4 Discussion

Children’s early outcomes have been shown to be related to their later educational performance (Aunio and Niemivirta, 2010; Bartik, 2014; Brownell and Drummond, 2020). While parental beliefs and behaviors play an important role in children’s early skill development (Lai et al., 2022; Sénéchal and LeFevre, 2002; Skwarchuk et al., 2014), previous studies have found some inconsistencies in those relationships (DeFlorio and Beliakoff, 2015; Missall et al., 2015). The aim of the present work was to examine the development of emerging language and math skills among Estonian five-year-old children in relation to their cognitive processes and learning skills and parental beliefs and behaviors. We assessed children’s skills directly by means of a nationally developed e-assessment instrument (Meesak et al., 2022, 2025) and asked parents to fill out a questionnaire about their perceptions of their children’s characteristics, home activities, involvement with the children’s kindergarten and expectations for their children’s skills. The study was conducted in a country recognized for its commitment to digital innovation and for children’s strong performance in early childhood (OECD, 2020b) and primary education (OECD, 2019; OECD, 2023), making it a relevant context for using innovative methods to examine children’s academic skills and the underlying relations with parental beliefs and behaviors.

First, we were interested in the relations between children’s skill areas (*RQ1*). As expected (*H1*), we found children’s language and math skills to be positively related to each other and to cognitive processes and learning skills. This is in line with previous studies, which have found relations between academic skills and cognitive processes (Best et al., 2011; Cameron et al., 2019) and learning skills (McClelland et al., 2006), showing the interplay of different early skill areas. At the same time, we found that the relation between children’s cognitive processes and learning skills might be more complex and mediated by other aspects. Similarly, previous research has shown that the relationship might not be as straightforward as between other skill areas (Meesak et al., 2022). This provides evidence for the importance of examining children’s development in a holistic way, covering various skill areas.

Second, we wanted to identify the constructs predicting children’s language and math skills (*RQ2*). We first looked at only child-assessed predictors and showed that 24% of the variance in children’s language skills is predicted by their own cognitive processes (attention and perception, mental flexibility and working memory) and learning skills (self-efficacy). Similarly, the same constructs help to explain 29% of the variance of math skills, with the additional construct of self-confidence. It should be noted that these results do not imply that the relationship between children’s cognitive processes, learning skills and early academic skills in unidirectional, rather that the relations are examined with a focus on academic skills. We then included parent-related predictors and found that their addition helped to increase the percentage of variance which can be explained in children’s language (35%) and math skills (32%), demonstrating the relevance of considering parental beliefs and behaviors when making inferences about children’s skills.

When it comes to language skills, in line with our hypothesis (*H2a*), children’s cognitive processes emerged as positive predictors as expected (Cameron et al., 2019; Morgan et al., 2019), alongside parental kindergarten-based involvement (OECD, 2020a; Puccioni, 2018), parental education (OECD, 2020a) and expectations of social skills. Even though the results of research by Puccioni (2018) did not show direct links between parental expectations regarding social and behavioral attributes and children’s academic skills, the results however indicated that parental expectations are related to their home activities, which in turn are related to children’s reading and math outcomes. Previous research has mostly concentrated on parents’ academic expectations of children’s skills (Liu and Hoa Chung, 2022; Skwarchuk et al., 2014), while the current research also highlights the importance of examining parents’ expectations regarding social skills. While perception of children’s cognitive difficulties was anticipated as a negative predictor (OECD, 2020a; Rutchick et al., 2009), home-kindergarten conferencing was also negatively related, suggesting that it has an adverse association when looking at multiple aspects concurrently. As the items on home-kindergarten conferencing focused on the extent parents discuss with teachers their children’s difficulties, peer relationships, and the need for doing more home activities, the results may stem from the fact that parents tend to communicate more when their children are already struggling. Even though children’s self-efficacy, self-confidence and parental perception of disruptive behaviors and expectations of learning skills were included in the model, they were insignificant. Notably, home activities and expectations of academic skills were entirely left out of the model, adding value to existing research, showing mixed results in these relationships (OECD, 2020a; Skwarchuk et al., 2014). In a previous study carried out with five-year-old Estonian children, only reading to a child at least five days a week was related to children’s language skills, while no other home activity had a significant effect (OECD, 2020a), supporting the notion that increasing the frequency of parental home activities might not always be most important, when looking at multiple aspects together.

In line with our next hypothesis (*H2b*), we found that children’s cognitive processes (attention and perception, mental flexibility, working memory), kindergarten-based involvement (positive predictors) and perceptions of cognitive difficulties (negative predictor) contributed unique variance to children’s math skills. These results complement previous research, which have also shown that parental perceptions of difficulties and kindergarten involvement are related to children’s academic skills (OECD, 2020a). In addition, children’s learning skills (self-efficacy and -confidence) emerged as significant predictors, highlighting the importance of developing children’s early learning skills, as brought out in previous studies (Doctoroff et al., 2016; McClelland et al., 2006; Yen et al., 2004). While parental social expectations predicted children’s language skills, similar relation did not emerge for math skills, which might reflect the inherently social nature of language development, which is more closely tied to interaction and communication (Vygotsky, 1978). Notably, once again, parental home activities did not emerge as significant aspects. While there are previous studies which have shown that five-year-old children’s math skills are related to home activities involving math (OECD, 2020a), there are also studies showing that parental home activities might not always be important in facilitating children’s academic outcomes (DeFlorio and Beliakoff, 2015; Missall et al., 2015). Considering the Estonian early learning context, where almost all five-year-old children attend kindergartens despite it being voluntary (Statistics Estonia, 2024), it can be deduced that parental home activities might not carry as much importance as they would in countries where children have less access to high-quality early childhood education.

Our findings emphasize the critical role of some child-assessed and parent-related predictors in shaping children’s academic skills. We bring attention to the importance of learning skills, which might be overlooked in most research, but contribute to academic skills development alongside cognitive processes. These results could be of value for kindergartens to consider the attention they are giving to five-year-olds’ skills, particularly their learning skills. In addition, active parental engagement (particularly through involvement in kindergarten activities), having higher expectations for social skills and perceiving fewer difficulties appear instrumental in fostering children’s academic competencies, while home activities might not be directly related to the development of these skills. While the current study made a distinction between parental language, math and social home activities, it should be noted, that similarly to previous studies (OECD, 2020a; Skwarchuk et al., 2014), the study concentrated only on the frequency of parental home activities, not their quality. These results can be used to inform parents to consider their perceptions of children’s difficulties and expectations of skills and to promote cooperation between parents and kindergartens.

The study had a cross-sectional design, which places some limitations on making inferences. While the relationships between children’s skills and parental beliefs and behavior were examined from the perspective of children’s academic skills, the design of the study does not allow for conclusions regarding the directionality or causality of these relationships. In interpreting the results, the context of the study setting should be taken into account. The study took place in a country with a relatively small and homogenous population, with equal opportunities for receiving preschool education (OECD, 2020a). However, drawing on sociocultural theory (Vygotsky, 1978) and related perspectives (e.g., Bandura, 1997), which emphasize that children’s learning and development are shaped by their cultural contexts, it is important to conduct research in countries that vary in size, language, culture and socioeconomic contexts. Such research is crucial for identifying the universal and underlying constructs of children’s learning, as well as describing culturally specific variations. While cultural and contextual differences must be considered when interpreting the results, the current research offers insights into children’s learning from a country with high-quality education and tendency for digital innovation. These findings may offer valuable perspectives for countries with similar educational aspirations; nevertheless, the results are not generalizable to broader or different populations. The study involved a recently developed e-assessment instrument for assessing children’s skills. The reliability of some constructs was lower than expected, but still acceptable taking into account the limited number of items (Cronbach, 1951) and possible variability of information gathered directly from young children. Even though the current study included children’s direct assessments, which should be preferred over indirect ratings (Kowalski et al., 2018; Vitiello and Williford, 2021), data from parents was gathered through a self-reported questionnaire. Future studies could benefit from including information from mothers and fathers separately (Fagan et al., 2024), though obtaining an equal sample might be difficult (Kikas et al., 2014). Future studies could also include more children’s skill areas, such as social and emotional skills, which are harder to assess with standardized testing. Although the study tried to achieve a representative sample with advanced sampling techniques, the final sample was modest, as the assessment mode was time-consuming, and some children did not participate in the study, while some parents did not fill out the questionnaire. Overall, our findings emphasize the multidimensional nature of the development of children’s academic skills and underscore the importance of considering both child and parental aspects in promoting early learning outcomes. Future research should further explore the dynamic interplay between children’s skills and parental influences to inform parents, teachers and researchers of the best ways to enhance children’s academic proficiency. Additionally, further research could employ qualitative methods alongside quantitative approaches to explore the depth and nuances of parental beliefs and behaviors. Mixed-methods research could provide richer insights into the quality and types of parental home activities, as well as beliefs that guide parental involvement in their children’s education.

## Data Availability

The datasets presented in this article are not readily available because, the study was part of a larger study conducted by the Estonian Education and Youth Board together with Tallinn University. The participants gave their informed consent for study participation and subsequent research, but consent for data publication was not obtained. Requests to access the datasets should be directed to the Estonian Education and Youth Board: https://harno.ee/.
