# Supplementary material for: Unlocking early academic skills: children’s cognitive processes, learning skills, and parental beliefs and behaviors predicting children’s language and math skills
Source: Front Psychol. 2025 Aug 20;16:1610243. doi: 10.3389/fpsyg.2025.1610243 (PMC12405427; doi:10.3389/fpsyg.2025.1610243)
Supplement: Supplementary file 1 [file Table_1.pdf]

Appendix A.

Table S1. *Model fit and comparison statistics for the questionnaire blocks.*

**Questionnaire block**	**Measurement model**							**Configural model/Metric model**				**Model comparison**		
	**CFI**	**TLI**	**RMSEA (90% CI)**	**SRMR**	**χ^2**	**df**	**p**	**CFI**	**TLI**	**RMSEA**	**SRMR**	**ΔCFI**	**ΔRMSEA**	**ΔSRMR**
Perceptions of children's characteristics	.95	.94	.04 (.03, .05)	.04	246.53	164	< .001	.91/.92	.90/.90	.06/.05	.06/.06	-.003	-.002	.002
Home activities	.96	.95	.05 (.02, .07)	.04	56.48	32	< .01	.95/.96	.93/.94	.05/.05	.05/.06	.002	-.003	.006
Kindergarten involvement	1.00	1.00	.00 (.00, .07)	.02	8.84	8	.36	.99/.98	.98/.97	.05/.06	.03/.05	-.01	.013	.017
Expectations of skills	.96	.95	.06 (.04, .08)	.05	101.35	41	< .001	.94/.93	.92/.91	.08/.08	.06/.08	-.01	.003	.025

Note: CFI = comparative fit index, TLI = Tucker-Lewis index, RMSEA = root mean square error of approximation, SRMR = standardized root mean square residual, χ^2 = Chi-square statistic.
