# Supplementary material for: Unlocking early academic skills: children’s cognitive processes, learning skills, and parental beliefs and behaviors predicting children’s language and math skills
Source: Front Psychol. 2025 Aug 20;16:1610243. doi: 10.3389/fpsyg.2025.1610243 (PMC12405427; doi:10.3389/fpsyg.2025.1610243)
Supplement: Supplementary file 2 [file Table_2.pdf]

Appendix B

Table S2. *Descriptive statistics for children's constructs and characteristics of parental beliefs and behaviors.*

	N	M	SD	Min	Max	Theoretical Range	No. of items
Children's constructs							
Attention and perception	279	20.42	2.23	13.5	23.5	0.00 – 23.50	7
Working memory	279	10.69	0.83	5.00	11.00	0.00 – 11.00	2
Mental flexibility	279	3.66	1.96	0.00	6.00	0.00 – 6.00	2
Interest	279	11.11	1.29	6.00	12.00	4.00 – 12.00	4
Self-efficacy	279	8.05	1.27	4.00	9.00	3.00 – 9.00	3
Self-confidence	279	8.46	1.08	3.00	9.00	3.00 – 9.00	3
Parental beliefs and behaviors							
Social difficulties	279	9.48	3.03	5.00	22.00	5.00 – 25.00	5
Cognitive difficulties	279	11.59	3.29	6.00	24.00	6.00 – 30.00	6
Learning ease	279	7.64	2.22	4.00	18.00	4.00 – 20.00	4
Disruptive behavior	279	8.25	2.41	4.00	16.00	4.00 – 16.00	4
Social home activities	279	17.86	3.23	6.00	25.00	5.00 – 25.00	5
Math home activities	279	9.86	2.63	3.00	15.00	3.00 – 15.00	3
Language home activities	279	6.48	2.12	2.00	10.00	2.00 – 10.00	2
Kindergarten-based involvement	279	8.37	2.57	3.00	15.00	3.00 – 15.00	3
Home-kindergarten conferencing	279	9.70	2.43	4.00	15.00	3.00 – 15.00	3
Academic expectations	279	22.03	2.55	9.00	25.00	5.00 – 25.00	5
Learning expectations	279	11.91	1.99	6.00	15.00	3.00 – 15.00	3
Social expectations	279	13.98	1.26	6.00	15.00	3.00 – 15.00	3
