# Supplementary material for: Unlocking early academic skills: children’s cognitive processes, learning skills, and parental beliefs and behaviors predicting children’s language and math skills
Source: Front Psychol. 2025 Aug 20;16:1610243. doi: 10.3389/fpsyg.2025.1610243 (PMC12405427; doi:10.3389/fpsyg.2025.1610243)
Supplement: Supplementary file 3 [file Table_3.pdf]

Appendix C.

Table S3. *Correlation matrix for child- and parent-assessed constructs.*

		2.	3.	4.	5.	6.	7.	8.	9.	10.	11.	12.	13.	14.	15.	16.	17.	18.	19.	20.	21
1.	Language skills	.60***	.42***	.21	.26**	.18	.15	.17	-.11	-.22*	-.06	-.06	-.02	.11	.12	.15	-.04	.19	.13	.25**	.21
2.	Math skills	1	.39***	.34***	.24*	.20	.20	.23*	-.10	-.23*	-.05	.02	.06	.15	.07	.14	.02	.12	.03	.10	.12
3.	Attention and perception		1	.17	.15	.15	.05	.13	-.05	-.07	.02	.05	-.06	.02	.02	.03	-.07	.05	-.09	.07	.05
4.	Working memory			1	.17	.14	.08	.08	-.03	-.14	-.11	.01	-.05	.05	-.09	.06	.06	.08	.01	.12	.11
5.	Mental flexibility				1	.13	.04	.04	-.10	-.16	-.07	-.06	-.02	.09	-.07	-.07	.08	.09	.18	.11	-.01
6.	Interest					1	.57***	.35***	-.23*	-.11	-.07	-.05	-.08	.08	-.07	.03	.07	.01	.05	.03	.06
7.	Self-efficacy						1	.34***	-.04	-.01	.02	.02	-.07	.10	.03	.03	.01	.03	.04	.03	.16
8.	Self-confidence							1	-.06	-.03	-.02	.01	-.04	.04	.05	-.03	.00	.00	-.02	.03	.05
9.	Social difficulties								1	.61***	.46***	.06	-.19	-.10	-.15	-.08	-.04	-.06	-.06	-.11	-.03
10.	Cognitive difficulties									1	.40***	.09	-.23*	-.25**	-.24*	-.12	-.03	-.12	-.01	-.02	-.03
11.	Learning avoidance										1	-.11	-.23*	-.12	-.16	-.14	-.11	-.12	.00	-.12	-.02
12.	Disruptive behavior											1	-.04	.02	.00	.07	.00	-.11	-.05	-.02	.07
13.	Social home activities												1	.36***	.45***	.17	.30***	.14	.01	.06	.01
14.	Math home activities													1	.38***	.01	.11	.26**	.01	.05	.01
15.	Language home activities														1	.13	.15	.20	.03	.10	.11
16.	Kindergarten-based involvement															1	.43***	.02	-.02	.09	.18
17.	Home-kindergarten conferencing																1	.04	.11	.09	-.01
18.	Academic expectations																	1	.19	.33***	.21
19.	Learning expectations																		1	.4***	.03
20.	Social expectations																			1	.23*
21.	Highest parental education																				1

Note: Constructs 1-8 are child-assessed, 9-20 are parent-related. Pearson correlation coefficients are shown (*** $p < .001$, ** $p < .01$, * $p < .05$). The p-values are adjusted by Holm's method.
